# An educational intervention based on family-centered empowerment model to modify high-risk behaviors of brucellosis via mother education

**DOI:** 10.1038/s41598-022-23385-5

**Published:** 2022-11-07

**Authors:** Maryam Sadat Shojaei, Seyedeh Belin Tavakoly Sany, Vahid Ghavami, Hadi Tehrani

**Affiliations:** 1 Department of Health Education and Health Promotion, Faculty of Health, Mashhad University of Medical Sciences, Mashhad, Iran; 2 Department of Health Education and Health Promotion, Student Research Committee, Mashhad University of Medical Sciences, Mashhad, Iran; 3 Social Determinants of Health Research Center, Mashhad University of Medical Sciences, Mashhad, Iran; 4 Department of Health Education and Health Promotion, School of Health, Mashhad University of Medical Sciences, Mashhad, Iran; 5 Department of Biostatistics, School of Health, Mashhad University of Medical Sciences, Mashhad, Iran

**Keywords:** Health care, Public health

## Abstract

The relative contribution of a theory-based intervention to modify high-risk behaviors in training programs is a major priority that remains an open question. Here, we tested whether the family-centered empowerment model used in the educational intervention was effective to modify high-risk behaviors of brucellosis via mother education. A quasi experimental study was conducted on 200 women presenting to healthcare practices in rural areas of Torbat-e Jam, Iran, from April 2020 to February 2021. Four rural areas were randomly assigned to the control and intervention groups. The intervention group received the training program, which included four 2-h sessions and consulting support via social network and messaging service. The control group did not receive any training. SPSS_16_ was implemented to test multiple statistical analyses. Our finding showed in the intervention group compared with the control group, knowledge, attitude, self-efficacy, self-esteem, and behavior outcomes were significantly changed (P < 0.001) across time during baseline through follow-up. Likewise, there are no differences (P > 0.05) in the change in construct of the family-centered empowerment model and risk behaviors in the control group from baseline to follow-up. Intervention based on a family-centered empowerment model is possible and very acceptable to modify high-risk behaviors of brucellosis by increasing an individual’s knowledge, changing attitude, and promoting self-efficacy and self-esteem.

**Trial registration**: Iranian Registry of Clinical Trials (IRCT), IRCT20160619028529N12. Registration date: 24/03/2020.

## Introduction

Brucellosis has been recognized as one of the most important zoonotic infections in the world with more than 500,000 human cases occurring annually in the world^1,2^. Brucellosis is still endemic in many parts of the world, including Africa^3,4^, Central Asia^5–8^, Mediterranean Basin^8–10^, Latin America^11^, and the Middle East where health systems rarely prioritize this infection^9,12^. Therefore, the World Health Organization (WHO) is considered this disease as a neglected zoonosis^13^. Brucellosis causes milk production decline, infertility, and abortion in cattle goats, swine, sheep, and dogs^8,14^. It is the most widespread zoonoses transmitted to humans through direct contact with infected animals or aborted fetuses, by consumption of contaminated meat and unpasteurized dairy products, or by inhaling airborne agents^4,14^. Therefore, farmers, ranchers, animal health personnel, laboratory personnel, abattoir workers, and other people involved in the livestock are the highest occupational risk groups. Human brucellosis has critical public health concerns and causes several symptoms like flue, including fever, fatigue, joint pain, weakness, sweating, malaise, and loss of appetite, weight loss, and arthritis^4,15^. It is often misdiagnosed as another infection disease, such as typhoid fever and malaria, resulting in underreporting and mistreatments^4,14^.With the rapid development of urbanization and animal industries, and the lack of appropriate hygienic scales in food handling and animal husbandry, brucellosis is mainly accounted as remaining a public health hazard^2,12,14^.

In the case of brucellosis, high-risk behaviors, including inappropriate behaviors such as consumption of unpasteurized dairy products, lack of personal protective equipment such as masks and gloves when working in the barn and warehouse, and contact with livestock secretions of infected animals, which are completely related to insufficient knowledge and awareness of people, their self-efficacy status and attitude^9,12^. Preventing high-risk behavior depends on increasing the level of awareness and improving the level of attitudes and beliefs, and a prerequisite for disease prevention is to have self-efficacy or the belief of people in their abilities to control risky behavior^4,6,14,16^. Since most livestock are kept mobile and there is no individual identification system, the eradication, and control of this infection may not be achieved by and test, slaughter, and vaccination only. In some regions, vaccination or eradication of infected livestock is not possible^4,6^. Therefore, prevention of human infection is primarily based on increasing occupational hygiene, food-safety measures, and safety knowledge^11,12^. Education training program about avoiding high-risk behaviors toward brucellosis (e.g., avoiding consuming contaminated meat and unpasteurized dairy products) and prevention strategies is important component to manage and control brucellosis^1,15,17,18^. Likewise, taking care of a patient with brucellosis is associated with several complications. Learning about brucellosis symptoms, transmission, and its adverse health effects as well as knowing about prevention strategies and treatment methods during treatment may cause many challenges for caregivers, among which is care burden^9,12,18^.

Family-centered care is a care philosophy that recognizes the importance of the family unit as the primary focus point in all health care. In fact, in this way, the patient's family can learn how to help their loved one who is suffering from an acute or chronic illness. The family-centered empowerment model is a theoretical framework that can reduce the level of care burden through increasing self-ability and improving mental and physical function^16,19^. This model is designed based on the effectiveness of the family members and individuals’ role on the three motivational, self-problem characteristics (knowledge, attitude, and perceived threat) and psychological (self-efficacy and self-esteem)^19–21^. The main target of the family-centered empowerment model is to strengthen the family members to improve the health level of the patient^19^. This model includes four steps, consisting first step: promoting the knowledge and attitude level through educational sessions. The second and third steps in improving self-efficacy and self-esteem through educational participation; and the fourth step, is evaluating all sessions during the empowerment training^19,21,22^. The family-centered empowerment model is used in several training programs to promote the care quality, reduce unfavorable side effects of a disease, and enhance patient and caregivers’ confidence^21^.

Although the data from the Ministry of Health and Medical Education the status of brucellosis in Iran is improving^2,12^, most parts of Iran are still endemic for brucellosis^17,18,23^. According to the epidemiologic data on trends of human brucellosis between 1991 and 2008, the yearly occurrence was around 43.24 patients per 100,000 populations^2,24^. In a recent study on 30 provinces of Iran, the mean incidence of brucellosis was 29.83 patients per 100,000 populations (45% females and 55% males and), and only 4.8% of the population were living in urban areas and 95.2% resided in rural areas^12,23^. Therefore, human brucellosis still remains a serious public concern in Iran and the situation of Iran among most other countries is troubling^12^. Although in Iran, livestock vaccination is provided free, the rate of livestock vaccination is low because of breeders’ insufficient awareness about brucellosis’s high-risk behaviors, consequences of untreated infection, transmission pathways, and prevention methods lead^24^. However, other factors, such as low individual knowledge about prevention methods, concerns about vaccine viability, inappropriate vaccination time and vaccine storage conditions, and inappropriate quarantine conditions mainly affect breeders’ preventive behaviors toward brucellosis^18,24^. To address this gap in the evidence base, this study aims to investigate the effectiveness of an educational intervention based on the family-centered empowerment model on modifying the high-risk behaviors of brucellosis through mother education.

## Methods

### Study design and participants

A quasi-experimental study examined the effect of an educational intervention based on a family-centered empowerment model on the management of high-risk behaviors in patients with brucellosis. We conducted this study from April 2020 to February 2021 in rural areas of Torbat-e Jam, Iran. Target population was mothers of patients with brucellosis who were supported by comprehensive rural health service centers in Torbat-e Jam. It was evidenced that there is a significant association between family health and mothering practices because the responsibility of family health falls mainly to the mother. Several studies reported that the mother has a key role in managing all aspects of family health and preventing disease and dealing with ill health^25,26^. It was assumed that mothers’ knowledge, attitude, and performance about managing brucellosis high-risk behaviors will be improved in the intervention group.

Therefore, we included mother of each family if they: (a) had a patient with brucellosis in their family, (b) living at least 6 months in the main centers of brucellosis, (c) and were aged 15 years ≤. Caregivers were excluded if they were: (a) unwilling to attend a training session and (b) had suffered types of disease and disability such as upper limb disability, visual impairment, and mental disturbance. The sample size were estimated based on the information about mean and standard deviation change of behavior scores between two groups, from a similar study by Babazadeh et al. who evaluated the efficacy of the family-centered empowerment model on the management of high-risk behaviors targeting patients with brucellosis^7^. Thus, we calculated the sample size equals to 60 participations in each group, using G-power 3.1.5 with consideration α = 0.05, $\beta$ = 0.2, effect size = 0.54 and 10% loss to follow up rate.

### Randomization and recruitment

**Figure 1:**
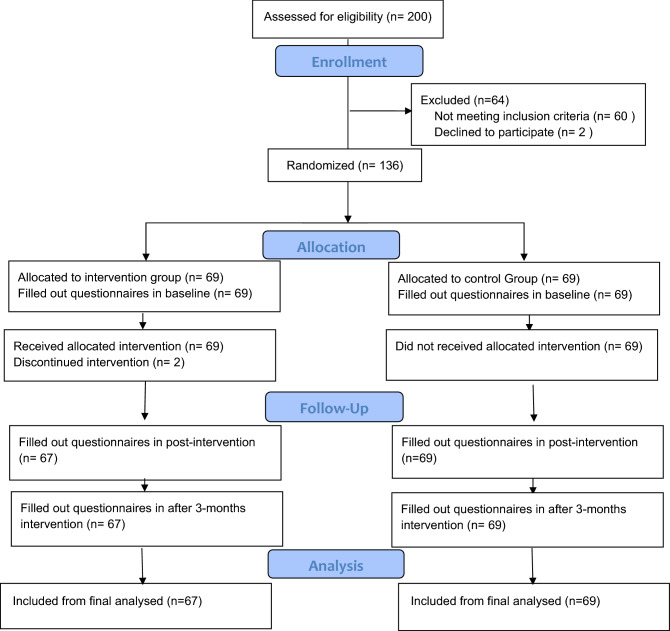
Flow of participants through each stage of the program.

Random allocation of healthcare centers was done based on the Consort-checklist and flow diagram (Fig. 1). All main centers of brucellosis in Torbat-e-Jam were listed according to the rural areas. Fours villages were randomly selected from a list and they were randomly assigned to the control (2 villages) and intervention (2 villages) groups. In each rural, we provided a list of mothers who had a patient with brucellosis in their family. Briefly, 200 mother were randomly selected, of whom 60 mothers have not meet inclusion criteria, and four participants refused to participate in follow-up due to the pregnancy, traveling, and employment problems. Finally, 136 mother were involved in the final analysis and they were assigned to the control (n = 69) and intervention groups (n = 67). All participants filled out a consent form and completed the baseline and follow-up process (Immediately after the intervention and 3 months after intervention) (Fig. 1).

### Intervention

We used the Template for Intervention Description and Replication checklist to conduct the intervention program^27^ (Table 1). The Family-centered empowerment model was used as a framework to design the educational program because it is a suitable social model to assess the determinants that influence an individual’s behavioral patterns and quality of life^21^.

**Table 1:** Template for intervention description and replication checklist in this study.

Items	Description
**BRIEF NAME**	Theory-based intervention for managing risk behaviors in patients with brucellosis
**WHY** (Rationale of treatment)	Brucellosis is a neglected tropical disease and it is associated with common zoonotic infection and morbidity among humans. Therefore, theory-based health education training is essential to control brucellosis. The main aim of this study was to examined the effectiveness of an educational intervention based on the family-centered empowerment model on modifying the high-risk behaviors of brucellosis through mother education
**WHAT (**Materials)	The intervention is informed by family-centered empowerment model, which is a suitable model for improvement of quality of life and health promotion. This model aims to empower the family system to promote its health with main focus on the effectiveness of the family members’ roles in three dimensions: motivational, cognitive and personal traits. The intervention targets key constructs of the family-centered empowerment model to promote cognitive characteristics (self-efficacy and self-esteem), and personal trait (knowledge, attitude, and perceived threat)
**WHAT** (Procedures)	This study included intervention and control groups. Experimental group received the full intervention program and consulting support. Control group did not receive any intervention. Four training sessions were scheduled with focus on the key constructs of the family-centered empowerment model that was explained with details in Table 2
**WHO PROVIDED**	A qualified training health educator and a physician provided training sessions
**HOW** (modes of delivery)	In the intervention group: four training sessions were conducted face to face by Lectures, questions and answers, brainstorming group discussion Pamphlets, Using teach-back video, review action planning reminder card and consulting support
**WHERE** (Infrastructure and relevant features)	Health promotion and Health education service in a public healthcare center
**WHEN and HOW MUCH** (Number of sessions, duration, intensity or dose)	Each participant engages 2-months training that included four 2-h training sessions (every 15 days)
**TAILORING** (Personalization)	Using lectures, questions and answers, educational pamphlets, and brainstorming group discussion, each session standardizing its time and strength
**MODIFICATIONS**	During the training, no rectification occurred to the organized intervention
**HOW WELL: planned** (Follow and procedure to preserve it)	For all participants, following the intervention was optimal
**HOW WELL: actual**	Without any deflection from the organized protocol, the complete (100%) planned intervention program was delivered

This model identifies multiple factors that empower the family members in promoting their health. Three dimensions for implementing this model have been identified includes, cognitive, personal traits, and motivation. Eligible mothers in the intervention group received a 2-month training that included four 2-h training sessions (every 15 days) with a focus on the key dimensions of the family-centered empowerment model, which are summarized in Table 2. Trainings were given in groups with 10 people and in the form of group discussions. In this study, we also considered consulting support by phone contact and social media to encourage caregivers to pay more attention to prevention strategies to manage and reduce high-risk behaviors of brucellosis. Consulting support were provided by the trainers for possible questions of the intervention group. A qualified training health educator and a physician provided training sessions and teach-back video, review action, educational pamphlets, and planning reminder card was used to conduct an intervention. In the control group, participants had not received any intervention, and they received a training package when the follow-up periods were finished.

**Table 2:** Family-centered empowerment model-based educational intervention to improve risk behaviors.

Times	Key constructs	Intervention method	Intervention’s targets
Sessions 1	Knowledge	Lecture (face to face), pamphlet, review memories, brainstorming group discussion	Clarified key concepts to promote individual’s knowledge**:** definition of the disease, types of **brucellosis,** potential risk factors, **transmission of brucellosis, symptoms,** potential prevention strategies, adverse health effect of **brucellosis, economic damage, treatment methods, and** vaccine-related knowledge
Sessions 2	Attitude	Lecture (face to face), pamphlet, review memories, brainstorming group discussion	Clarified expected benefits of vaccination and preventive strategies (such as prevention of **brucellosis** diseases, slaughter practices, benefits of pasteurized or boiled milk consumption decreased adverse health effect**,** and **economic damage**) to improve individual’s positive attitude and belief to increase their commitment towards prevention strategies and practice Asked to explain the reasons for the prevalence of brucellosis from their point of view, personal experiences with the disease, **symptoms of brucellosis** that they or their family have experienced, and type of cooperation in the treatment process of their family members Encouraged to share your experiences and knowledge with your family about this disease
Session 3	Self-efficacy and self-esteem	Lecture, pamphlet, question and answer, barrier identification, brainstorming group discussion	Promoting individual capability and self-confidence to promote desired healthy behavior based on: recognize early symptoms, refer a doctor in a timely manner, adhering medicine, Proper and regular use of medication Explained how to improve the strength of the participants’ belief in their ability to engage in preventive behaviors and practice on a regular basis despite various conflicting situations such as do not consume raw meat and local dairy products, using protective safe equipment when working in livestock, timely vaccination of livestock, providing suitable and hygienic environment for keeping cattle Identified barriers perceived as preventing women from performing preventing practices and teach how overcome perceived barriers: for example, personal (lack of knowledge and skills and lows elf-efficacy), economy and environmental barriers
Session 4	Modifying high-risk behaviors	Lecture, pamphlet, brainstorming group discussion,	Increase awareness and commitment to desired behavioral endpoint or outcome: Familiar with high-risk behaviors associated with brucellosis infections (avoiding the consumption of uncooked meat and raw milk, appropriate preserving animal-based food) Familiar with protective measures (wearing gloves when delivering or handling abortion materials and vaccine)
From session 1 to session 4	Counselling support	Group discussion, questions and answers, teach-back video	Help to set and review personal goals and prevention strategies to overcome barriers and act health behaviors

### Measuring tools

This study examined the effect of an educational intervention based on a family-centered empowerment model on the management of high-risk behaviors in patients with brucellosis. The outcome of this study was to reduce high-risk behaviors toward brucellosis based on changing cognitive characteristics (self-efficacy and self-esteem), and personal traits (knowledge, attitude, and perceived threat). The intervention based on family-centered empowerment model was considered as an independent variable, and the covariates studied were demographic factors including age, marital status, occupation, education, income, and household size, and history of brucellosis in family members.

The research questionnaires used in the study were as follows: demographic data questionnaire (14 items), high-risk behaviors questionnaire (17 items), and the empowerment evaluation questionnaire (37 items) that included questions on awareness, attitude, perceived threat, Rosenberg’s self-esteem and self-efficacy in high-risk behaviors related to brucellosis.

In this study, we used the family-centered empowerment model as a framework to design a questionnaire. To design the items that related to the family-centered empowerment model and high-risk behaviors, we studied the relevant literature (12) and interviewed 30 caregivers to collect their opinions concerning high-risk behaviors related to brucellosis. Then, an expert panel of ten specialists in health education, epidemiology, and physicians edited assessed the relevance and necessity of all questions to estimate the content validity index (CVI) and content validity ratio (CVR). In this study, the average CVR for the high-risk behaviors questionnaire and the family-centered empowerment questionnaire was 0.88 and 0.98, and CVI for these questionnaires was 0.94 and 0.92, respectively. We examined clarity, readability, and simplicity of questions based on the pilot which included 50 caregivers. The reliability coefficients (Cronbach’s α) of the high-risk behaviors questionnaire and the empowerment evaluation questionnaire were 0.88 and 0.85, respectively, suggesting an acceptable internal consistency of the tool’s criterion (20). Likewise, the appropriate correlation coefficient between all constructs of family-centered empowerment was observed.

#### Family-centered empowerment questionnaire

The empowerment evaluation questionnaire included questions on changing personal traits (knowledge, attitude, and perceived threat) and cognitive characteristics (self-efficacy and self-esteem). Knowledge about brucellosis was examined by 15 items based on information related to potential risk factors, the transmission of brucellosis in humans, symptoms, and as potential prevention strategies for disease (e.g., Raw and undercooked meat can be effective in transmitting malt fever?), which were ranged on a 3-point scale from 1 (false option), 2 (I don’t know), and 3(correct option). Cronbach’s α of this construct was 0.75. We used 12 items to estimate attitude, and perceived threat (e.g., I believe that livestock vaccination is important to prevent brucellosis in humans, or if I get sick, brucellosis has no side effects for me). All questions were ranged on a three-point scale ranging from 1 (disagree) to 3 (agree) and achieving a higher score meant a more positive attitude. Cronbach’sαon this scale was 0.93. The self-efficacy contained 7 questions (e.g., If I get sick, I can take my medication properly? Or I vaccinate my cattle, even if they cost a lot (with a three-point scale ranging from 1 (disagree) to 3 (agree) (23). Cronbach’sα on this scale was 0.97. Rosenberg’s self-esteem was measured by ten-question (e.g., I feel like a valuable person, at least equal to others?), which were evaluated based on a two-point scale from 1 (disagree) to 2 (agree). Cronbach’s α on this scale was 0.84.

#### High-risk behaviors

Prevention strategies to manage and reduce high-risk behaviors of brucellosis was examined by 17 questions on factors such as methods of processing milk, dietary habits, husbandry practices, environmental sanitation, and dairy products (e.g., I use masks and gloves when working in the barn and cleaning livestock or I use freshly packaged milk). Items in this questionnaire were rated from 1 (never) to 5 (always), and Cronbach’s α on this scale was 0.99.

### Statistical analysis

Data analysis was conducted using the descriptive analysis (mean and standard deviation) and bivariate analyses (chi-square, independent and paired t-tests, and Fischer’s test) to examine variation between different variables in control and intervention groups. We used the repeated measure ANOVA to estimate differences in a different outcome from baseline to follow-up in both groups. The generalized estimating equations (GEE) were also used to examine the effectiveness of the intervention on improving the high-risk behaviors in study population. Likewise, this analytical model allows for the accounting of cluster effects between model constructs (constructs of knowledge, attitude, self-efficacy, self-esteem) and behavior outcomes within an intervention program. We assumed training programs to be nested within improving high-risk behaviors via the Family-centered empowerment model’s constructs. This analytical analysis engaged adjustment for covariates that significantly affected outcomes because the main objective in this survey was examining the effectiveness of the family-centered empowerment model and its constructs on improving high-risk behaviors toward brucellosis. The estimated model constructs for the determinants of high-risk behaviors are accompanied by their 95% confidence interval (CI) and P < 0.05 were considered as significant differences. Data analysis was conducted by SPSS_16_.

### Ethics approval and consent to participate

This article research project approved by Ethics Committee of Mashhad University of Medical Sciences (IR.MUMS.REC.1398.199) and was conducted with consideration of Helsinki Declaration in all phases of the study. Confidential data treatment was guaranteed. Written informed consent was obtained from the participants. Availability of data and materials Data from this study will not be openly available until planned publication outputs have been completed.

## Results

We conducted a quasi-experimental study with 136 mothers. The socio-demographic characteristics of the study participants are summarized in Table 3. There were no statistically significant differences in these characteristics from control and intervention groups (P > 0.05). Our finding showed that the mean (± SD) of age and family number for the enrolled participants were 38.02 (± 11.32) and 4.16 (± 1.62), respectively. A majority of the eligible participants were married (94%), housekeepers (94.5%), low-income families (96.2%), and literate (63.7%) (Table 3).

**Table 3:** Participant’s socio-demographic characteristics.

Variables	Intervention group (*n* = 67)	Control group (*n* = 69)	Statistical test result*
Age, *years, m* ± *SD*	37. 64 ± 12.78	38.19 ± 11.94	0.79
Household dimension	4.19 ± 1.73	4.67 ± 1.84	0.89
**Mother education, *%***			
Illiterate	27(40.3)	21 (30.4)	0.22
Literate	40 (59.7)	48(69.6)	
**Spouse’s education**			
Illiterate	21(33.3)	20 (28.1)	0.52
Literate	46 (66.7)	49 (71.9)	
**Marital status, %**			
Married	63 (94)	64(92.8)	0.19
Widow or divorced	4(6)	5(7.2)	
**Occupation, *%***			
Housewife	62 (92.5)	67 (97.1)	0.47
Employed	5 (7.5)	2 (2.9)	
**Spouse’s occupation, *%***			
Farmer or rancher	45(68.1)	39 (56.1)	0.171
Other jobs	22 (31.9)	30 (43.9)	
**Income, *%***			
Low	65(97)	65(94.5)	0.68
Moderate	2(3)	4 (5.8)	
**Spouse’s income**			
Low	48 (72.2)	52 (78.3)	0.48
Moderate	19(27.8)	17(21.8)	
**History of brucellosis in family**			
Yes	47(70.1)	54(78.3)	0.27
No	20(29.9)	15(21.7)	
**History of brucellosis in participants**			
Yes	38 (56.7)	36 (52.2)	0.595
No	29 (43.3)	33 (47.8)	

*N* number of eligible participants; ± standard division; *Testing significant change between control and experimental groups which is significant at the 0.05 level.

The results of comparing participants’ knowledge, attitude, self-efficacy, self-esteem, and behavior outcomes in control and intervention groups in all-time points (baseline, immediately after intervention, and 3-months follow-up) are explained in Table 4. The results showed that there was no significant difference (P > 0.05) between scores of the family-centered empowerment model’s constructs in control and intervention groups at baseline. Our finding showed that among the participants in the intervention group compared with the control group, knowledge, attitude, self-efficacy, self-esteem, and behavior outcomes were significantly changed (P < 0.001) across time during baseline through follow-up (Table 4). Likewise, there are no significant differences (P > 0.05) in the change in construct of the family-centered empowerment model and risk behaviors in the control group from baseline to follow-up. Thus, the time effects for all constructs were different in the intervention group as compared to the control group. The score of all measurements was significantly increased immediately after intervention (P < 0.001) In the intervention group; but, these scores were decreased overtime at 3 months after the training. In control groups, the time trend was not significant for all constructs (P > 0.05). Based on the results of covariance analysis, the educational intervention had a significant effect on all constructs of the model for all post-intervention measurements and follow-up measurements. Therefore, the training program in this study has led to an increase in the score of participants’ knowledge, attitude, self-efficacy, self-esteem, and behavior outcomes in all measurements after intervention (Table 4).

**Table 4:** Average scores for family-centered empowerment questionnaire’s constructs from baseline to follow up in control and intervention groups.

Model’s constructs	Pre-intervention					Post-intervention					3-month follow-up					From prev. follow-up	
	Intervention		Control		*P value	Intervention		Control		*P value	Intervention		Control		*P value	Intervention	Control
	Mean	SD	Mean	SD		Mean	SD	Mean	SD		Mean	SD	Mean	SD		**P value	**P value
Knowledge	35.90	4.30	37.20	3.64	0.055	43.26	1.26	37.17	3.91	< 0.001	42.23	1.59	36.66	4.25	< 0.001	< 0.001	< 0.62
Attitude	27.97	4.43	26.82	4.79	0.158	34.13	1.74	26.53	4.38	< 0.001	33.46	1.96	26.01	4.48	< 0.001	< 0.001	< 0.11
Self-efficacy	18.05	2.75	18.91	2.46	0.065	21.32	1.05	18.68	1.90	< 0.001	21.01	1.22	18.21	2.35	< 0.001	< 0.001	< 0.13
Self-esteem	18.38	1.72	17.54	1.84	0.2	20.68	0.72	17.36	1.90	< 0.001	20.46	0.82	17.14	1.91	< 0.001	< 0.001	< 0.16
Risky behavior	54.14	12.62	58.17	13.80	0.139	73.01	8.78	57.92	12.41	< 0.001	70.97	8.78	56.78	12.69	< 0.001	< 0.001	< 0.21

*n* number of eligible participants, *SD* standard division; *Testing significant change between control and intervention groups; **Testing significant change from pre-intervention to 3-month follow-up.

This result was synchronous with the GEE models that provided significant interaction effects between group and time. The results of this model showed a significant improvement (P < 0.001) in the change in behavior outcome among the participants in the intervention group compared with the control group across time during baseline through follow-up. Likewise, this model showed that among the all constructs of family-centered empowerment, only the attitude construct had a significant direct association (P = 0.019, 95% CI (0.112, 1.4.8)) with behavior outcome (Table 5).

**Table 5:** Effectiveness of the intervention on improving the high-risk behaviors via model constructs in different group and time period.

Variables	The regression coefficient	95% CI	*P value
**Group**			
Intervention	11.594	7.512 to 15.676	< 0.001
Control	0	–	–
**Time**			
Two months after the intervention	− 0.896	− 1.311to − 0.482	< 0.001
Immediately after the intervention	0	–	–
Awareness	0.229	− 0.149 to 0.607	0.235
Attitude	0.760	0.112 to 1.408	0.021
Self-efficacy	− 0.162	− 0.956 to 0.631	0.689
Self-esteem	0.272	− 0.586 to 1.131	0.534

*CI* confidence interval; *Testing significant effect between groups and time period.

## Discussion

Human brucellosis is still considered a major zoonotic infection with a high frequency in many provinces of Iran^12,26^. Therefore, the implementation of a national brucellosis control program to promote awareness of brucellosis and preventive behaviors among occupational workers and livestock is an important aspect of brucellosis control in both animals and humans^24^. The purpose of this study was to evaluate the effectiveness of an educational intervention based on the family-centered empowerment model on modifying the high-risk behaviors of brucellosis through mother education. In fact, in this study, we tried to empower the family members to modify their risky behaviors toward brucellosis.

According to the results of this study, the family-centered empowerment model-driven educational intervention significantly modified mothers’ high-risk behaviors toward brucellosis by increasing their knowledge, changing attitudes, and promoting self-efficacy and self-esteem. Our finding confirms that mothers in the intervention group, under theory-based intervention, behaved better in preventing brucellosis and reducing brucellosis incidence in their families. Some studies recently investigated the effect of theory-based educational intervention in the adoption of appropriate behaviors and modification of high risky behaviors to prevent brucellosis in humans and livestock^8,15,18,26^. They mainly planned the educational interventions based on participants’ experience and knowledge or used other models to behavior change, such as PRECEDE-PROCEED^18,23,26^ and Health Belief Model (HBM)^13,17^. Most of these studies highlighted educational theories or models are effective frameworks that can guide providers to truly evaluate the risk of infection and identify preventive methods that can reduce and control the prevalence of the disease^13,18,23,26^.

However, it was evidenced that educational theories or models are not “culturally versatile” and do not account for socioeconomic characteristics and cultural factors of the target population^18,23,26^; therefore, several studies recommended conducting the same studies in different socioeconomic and cultural contexts to examine the power of these theories to change behaviors^11,13,17,18^. Our study is in line with their recommendations that emphasize the interdisciplinary effective survey, such as the combination of health education and brucellosis prevention strategies” to improve the prevalence of brucellosis and its burden in different endemic regions^1,14,18^. To the best of our knowledge, there is no training program based on a family-centered empowerment model that has examined the combination of health education and brucellosis prevention strategies. In this study, the family-centered empowerment model, as one of the main participatory health models^21^ which has been used to design training programs, successfully provides a clear appropriate framework to design a participatory intervention program. The significant difference in the level of risky behaviors toward brucellosis in the intervention group compared to the control group confirmed the efficacy of the family-centered empowerment model in modifying high-risk behaviors of brucellosis. Therefore, it is recommended to use such training on improving the health and risky behaviors of livestock breeders and their families through longitudinal studies.

Knowledge and awareness-related behavior is the main construct of the family-centered empowerment model that influences individuals’ practice. In this study, at baseline, the participants lack adequate knowledge and appropriate experience in safe behaviors toward brucellosis. All participants had heard of brucellosis, its zoonotic nature, and what the government was doing regarding brucellosis (vaccination and namely control), but they did not have inadequate and superficial knowledge regarding the recognizing symptoms of brucellosis in humans and animals, mode of transmission, and prevention methods. Poor knowledge scoring was also reported in several studies that evaluate the individuals’ knowledge of brucellosis in Africa^3,4^ and Asia^3,6,8,26^, which are the endemic regions of brucellosis. They reported that only approximately half of the population (including livestock and their family members, resident, and other occupation-related groups) knew about brucellosis^4,12^, which confirms the insufficient level of awareness and knowledge of brucellosis in this region. In Iran, several factors have been identified as main contributors to the low level of brucellosis knowledge is among the livestock value chain such as insufficient knowledge-creating activities provided by veterinary departments and public health agencies^12,24,26^, low literacy and self-efficacy rates^15,17,18^, and little training on the handling and rearing of animals^7,13,17,26^, and a lack of health education programs^5,18,25^. Our finding also showed that the score of mother’s knowledge was significantly increased immediately after intervention in the intervention group. This might be because of correct training that was provided to identify key concepts to avoid the high-risk behaviors associated with brucellosis. Several studies have indicated that raising the brucellosis-related knowledge is the main aspect that led to a higher stage of change and commitments to modify risky behaviors of brucellosis and inadequate knowledge leads to neglect in disease prevention and treatment^4,14,23^. Therefore, the implementation and development of brucellosis control programs and more efficient health educational intervention that should fit the perception and needs of the local population are necessary to improve the knowledge and control of brucellosis.

In this study, the mothers in the intervention group showed a greater change in the average score of attitude, as compared with the control group at the follow-up and post-intervention. Likewise, our finding highlighted the role of attitude as the most important determinates on behavior outcome. This might be due to the effect of educational intervention that was clarified the expected benefits of vaccination and preventive strategies associated with brucellosis. In the intervention group, we provided correct information on preventive strategies that is practical to equip individuals in society to safeguard themselves. We also asked participants to share their points of view and personal experiences regarding disease transmission and symptoms. This made respondents show a very positive attitude and willingness to receive more information on brucellosis prevention and treatment. A positive attitude toward the importance of chronic disease in cattle and especially abortion lead to better identification of disease in livestock^3,4^. It was evidenced that raising the brucellosis-correct information is the main aspect that has influenced their willingness and ability to modify risky behaviors of brucellosis^3,4,6,17^. Therefore, it is critical for the relevant healthcare professionals and authorities to take note of positive attitude as the main influential factors to modify risky behaviors of brucellosis.

Similarly, the intervention had significant effects on increasing self-efficacy and self-esteem in the intervention group when compared with the control groups, which was consistent with the results of other surveys using the family-centered empowerment model^20,21^. They highlighted the effect of self‑efficacy on modifying risky behaviors of brucellosis in rural mothers^7,18,21^. Self‑efficacy is defined “as the perceived ability to perform a target behavior and refers to positive and negative feelings and people's judgments about their ability to master the situation”^28^. During the training sessions, all mothers learned how to set realistic goals to improve their ability to engage in desired preventive behaviors and practice (e.g. avoiding eating fresh meat and cheese, boiling milk, timely vaccination of livestock, and using protective safe equipment) despite various conflicting situations. We also identified barriers and teach them how to overcome perceived barriers via precise and simple which is leads to creating a sense of self‑efficacy and empowerment in participants. Our result supports other studies that found an increase in the individual's self-efficacy and self-esteem could be made higher stage of commitment to modify healthy behaviors^7,17,18,21^.

### Strengths of the study

Our study is one of the first to examine the effectiveness of an educational intervention-based family-centered empowerment model on brucellosis control and prevention strategies in Iran as the main endemic region of brucellosis in Asia. Testing the application of the educational theories and practices in different communities is essential to improve the theory-based intervention and understand the crucial determinates in the successful training program. A theory-based intervention like the present research tests the power of mediator’s constructs and finds out how these constructs influence an individual’s abilities and attitude to commit to specific behaviors. The findings of this study highlight the importance of theory-based intervention in local settings to modify community’s high-risk behaviors by promoting their knowledge and self-efficacy through utilizing their positive attitudes.

Likewise, in this study, the positive effects of the intervention based on the family-centered empowerment model on successful behavior changes were sustained because the mean scores of mediator constructs in the intervention group were still higher than the mean of these scores in the control group at the follow-up. This could be related to useful theoretical mediator constructs (includes knowledge, attitude, self-efficacy, and self-esteem) that were used in this training program. The content of the questionnaire and educational sessions were tailored based on situational and need assessment. However, the declining trend of the scores in both groups after 3 months reminds the need for continuous. Results from this public health intervention will provide a basis for future educational training aimed at modifying the high-risk behaviors toward brucellosis. It is an acceptable and feasible educational intervention as needs minimal time and investment by the training of rancher family members or occupationally exposed populations.

### Limitations

This study is subject to some sources of limitations in the process of design and implementation of the theory-based intervention. Most of the participants had a low level of education or were illiterate. To overcome this bias, we explained and read all items of the research questionnaires for participants so that they could complete the questionnaire. Self-reporting is another limitation in our study that may affect the outcome of investigations. Likewise, there is the possibility that some mediator questions in research questionnaires may not reflect all aspects of the family-centered empowerment model’s constructs in a natural setting. However, the probability of this bias is unlikely to occur because the result of test–retest reliability was more than 0.9 for research questionnaires in this study. In addition, some questions may not be sensitive enough to detect all changes during follow-up because the multiple preventive behaviors were targeted in the training program. Therefore, further studies on family-centered empowerment models are still needed to test its specific change constructs in different populations. Identification of successful mediators constructs to maintain stable behavior are part of a field that needs further investigation via theory-based interventions in the future.

## Conclusion

This study contains useful findings to understand the brucellosis control and prevention strategies. International evidence indicates brucellosis requires more attention, and intervention strategies based on behavior change theory are the main method to manage and control brucellosis. Our finding highlighted the family-centered empowerment model-driven educational intervention significantly modified mothers’ high-risk behaviors toward brucellosis. Likewise, the utility of family-centered empowerment model constructs for modifying the high-risk behaviors of brucellosis was supported since individuals in an intervention group moved to a higher stage of change if they had higher knowledge, attitude, self-efficacy, and self-esteem. It appears that the use of family-centered empowerment model and its potential constructs could be used as a promising educational theory to develop behavior change success. Therefore, existing educational sessions between cattle keepers and government veterinary services could be utilized optimally to provide relevant information on human and livestock health, brucellosis control, and prevention strategies. Raising the brucellosis-related knowledge, changing attitude, and promoting self-efficacy and self-esteem would probably be more efficient to modify risky behaviors of brucellosis if provided through the appropriate training program, which in this study was conducted based on a family-centered empowerment model. Likewise, more attention should be paid to improving brucellosis-related knowledge and attitude of the rancher or occupationally exposed populations and to ensure that caregivers are equipped to treat this zoonotic disease.

## Data Availability

Datasets used and/or analyzed during the current study are available from the corresponding author on reasonable request.

## Abbreviations

WHO: World Health Organization
CI: Confidence interval
SPSS16: Statistical Package for Social Sciences software
GEE: The generalized estimating equations
HBM: Health Belief Model
